# Large Intermuscular Lipoma Presenting as a Groin Hernia

**DOI:** 10.7759/cureus.11584

**Published:** 2020-11-20

**Authors:** Roneil N Parikh, Fadie Aziz, Damacent Rutagengwa, Auerilius E Hamilton

**Affiliations:** 1 General Surgery, Campbelltown Hospital, Sydney, AUS; 2 General Surgery, Liverpool Hospital, Liverpool, AUS; 3 General Surgery, Western Sydney University, Campbelltown, AUS; 4 Colorectal Surgery, Campbelltown Hospital, Sydney, AUS; 5 Macarthur Clinical School, Western Sydney University, Campbelltown, AUS

**Keywords:** lipoma, intermuscular lipoma, inguinal hernia

## Abstract

Lipomas are the commonest benign mesenchymal tumours, commonly seen as a subcutaneous lump. However, intermuscular lipomas are uncommon and can remain asymptomatic until they attain larger sizes. We discuss a rare case of a large symptomatic intermuscular lipoma in a 34-year-old woman who presented with acute on chronic groin pain. Clinical examination findings were consistent with an incarcerated inguinal hernia and imaging confirmed a large intermuscular lipoma of the anterior abdominal wall, the tip of which herniated into the inguinal canal. She underwent open primary repair of the hernia along with excision of the lipoma. An intermuscular lipoma presenting as an incarcerated inguinal hernia at the first instance is an uncommon finding. Due to lack of obvious clinical findings, uncomplicated intermuscular lipomas can be challenging to diagnose until they become symptomatic, and a high degree of suspicion in patients reporting atypical abdominal and groin pain, or abdominal wall fullness is required.

## Introduction

Lipomas are the commonest benign mesenchymal tumours originating from the subcutaneous adipose tissue, usually presenting as soft subcutaneous lumps. They are usually located superficial to the enclosing fascia in the subcutaneous tissues, however, they may be localised deep to the enclosing fascia and are called deep-seated lipomas [1]. They can either be found between the muscles (intermuscular) or within the muscles (intramuscular). Both of these are rare with an incidence of 0.3% and 1.8% respectively of fatty tumors, seen predominantly in the middle-aged and older population [1-3].

Intramuscular lipomas are found most commonly in the lower extremities, with the trunk being the next most common, whereas intermuscular lipomas most commonly affect the anterior abdominal wall [4,5]. Intramuscular lipomas may be divided into well-circumscribed and infiltrative types. Well circumscribed lipomas have a distinct boundary, are capsulated, and easily distinguished from the adjacent muscles. Infiltrative lipomas invade muscle fibres and eventually replace them [3,5].

Due to their deep location, intramuscular and intermuscular lipomas are often large at diagnosis and rarely may present as hernias, which may only represent the “tip of the iceberg” [6]. A delayed presentation of these lipomas may increase the risk of de-differentiation [6]. Therefore, a high index of suspicion, timely diagnosis, and careful surgical excision are required to treat them. We highlight a case of a symptomatic intermuscular lipoma in a 34-year old woman presenting acutely as a symptomatic inguinal hernia.

## Case presentation

A 34-year-old woman presented to our emergency department with nausea, dry retching and severely worsening right groin pain. Over six months, she had recurrent right lower abdominal burning pains localised to the right groin, which was now aggravated by moving and straining. She did not have any vomiting or obstipation suggestive of a bowel obstruction. Her previous surgical history was only a lower Pfannenstiel incision abdominoplasty done two years prior. Apart from occasional constipation, she had no significant medical history, traumatic injuries, or metabolic disorders.

**Figure 1:**
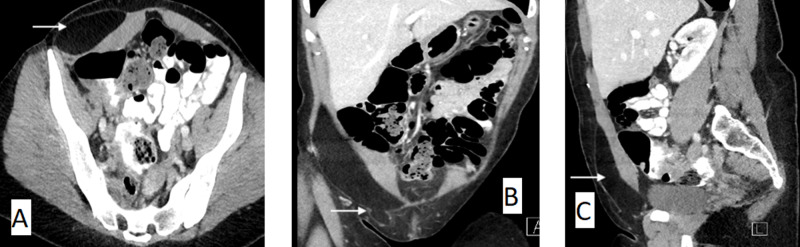
Axial (A), coronal (B), and sagittal (C) view of a CT scan of the abdomen and pelvis with arrows demonstrating a large right anterior abdominal wall lipoma arising between the external and internal oblique muscles extending into the inguinal canal.

A groin ultrasound organised by her general practitioner few days prior reported an “indirect right-sided inguinal hernia with a 10mm neck, and sac measuring 30mm x 29mm x 9mm containing adipose tissue, reducible with probe pressure.” In ED, she had normal vital signs and was afebrile with a soft non-distended abdomen. She however had localised right groin tenderness and voluntary guarding over an irreducible lump without an obvious cough impulse or overlying skin changes. Blood investigations showed normal septic parameters. A contrast-enhanced abdominal CT scan was organised by the emergency department to exclude other acute intra-abdominal pathology, prior to surgical referral. The CT confirmed a large right-sided 80mm x 80mm x 130mm abdominal wall lipoma lying between the external and internal oblique muscles. The inferior aspect of this lipoma “appeared to herniate into” the inguinal canal with nothing herniating from the intrabdominal cavity (Figure 1).

**Figure 2:**
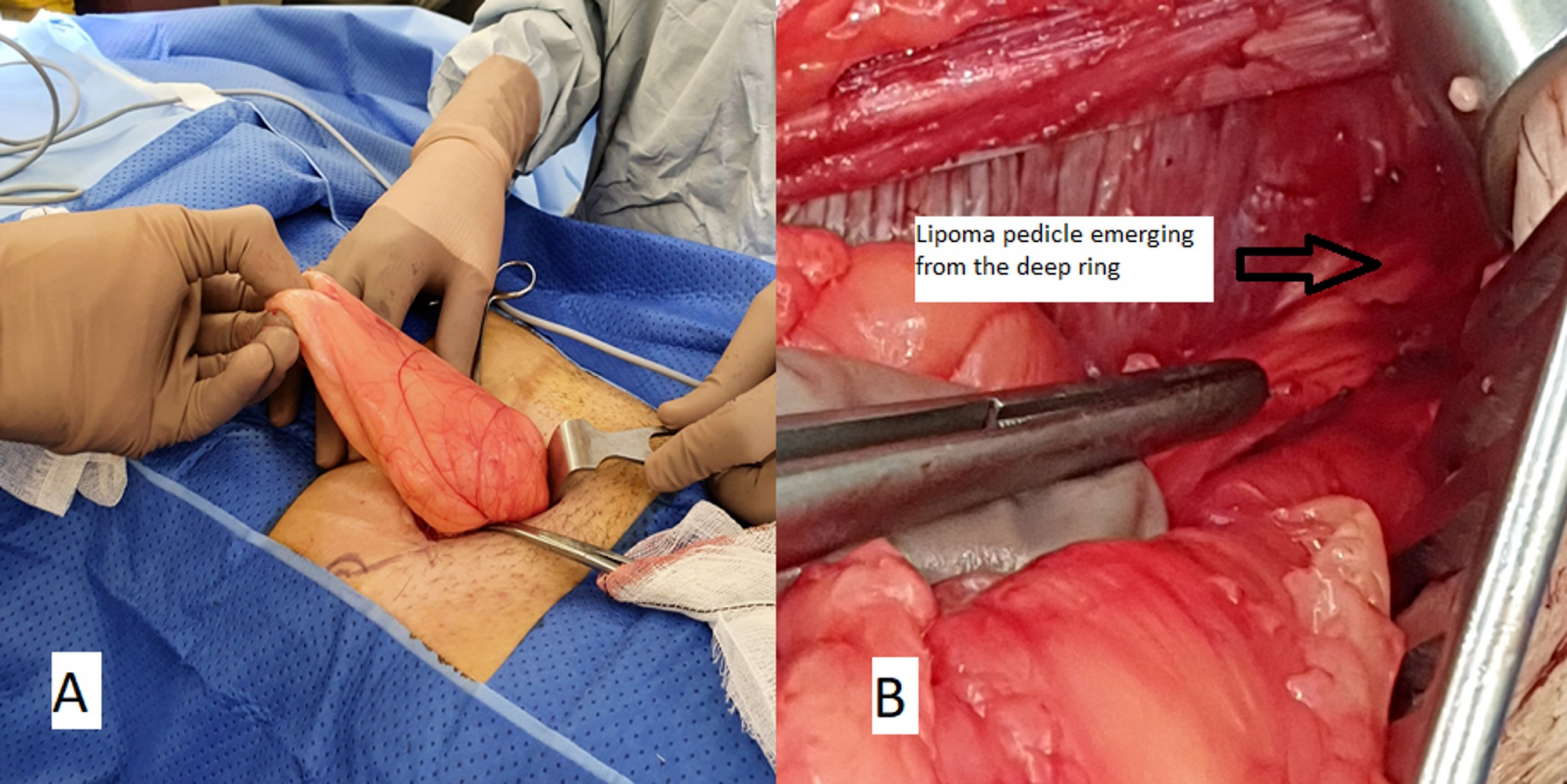
Intraoperative image during open inguinal hernia repair demonstrating the right groin with the lipoma being extracted with an intact capsule from the groin incision (A). Lipoma was dissected from the intermuscular plane and was attached by a pedicle at the internal ring demonstrated by an arrow (B).

**Figure 3:**
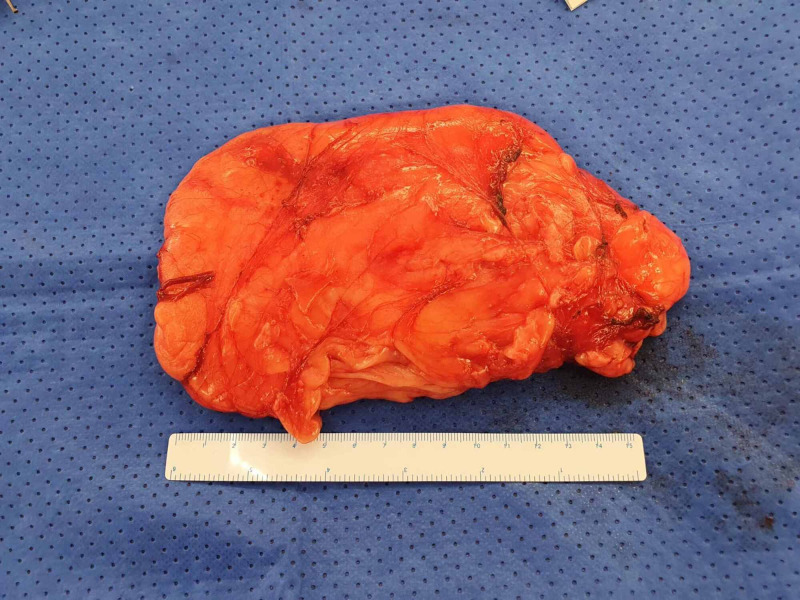
Excised specimen demonstrating a large well-circumscribed mass measuring 160mm x 90mm.

When reviewed by the surgical team, the sudden worsening of her pain combined with the above clinical examination and imaging findings was suggestive of a now irreducible fat-containing inguinal hernia. Due to ongoing pain, she underwent open inguinal hernia repair through part of the Pfannenstiel incision scar. At operation, the large lipoma was easily enucleated from the intermuscular space, however, it had grown under the ilioinguinal nerve which was now being stretched by the lipoma. The vascular pedicle to the lipoma arose through the internal ring, where it was then ligated (Figures 2, 3).

The small hernia defect was primarily repaired with nylon sutures, as a synthetic mesh was deemed unnecessary and at a high risk of infection should a seroma develop. She was discharged home the next day with an uneventful recovery. Subsequent histology findings demonstrated mature adipose tissue, without any evidence of malignancy, confirming the diagnosis of a simple lipoma.

## Discussion

Although subcutaneous lipomas are common, intermuscular lipomas consist of only 1.8% of all soft tissue tumours. When located over the anterior abdominal wall, the lump may not be obvious until the lipoma attains a large size [2]. The larger sized lesions can herniate through the abdominal wall or groin, however, the herniating part may only represent the “tip of the iceberg” and the remainder still undetected [6].

It is important to recognize these because of their differential diagnosis of liposarcomas. They are differentiated from lipomas histologically by their atypical nucleus, mucous degeneration, polymorphism, and mitosis [5,7].

This unusual intermuscular lipoma likely caused pain over few months as it grew under and stretched the ilioinguinal nerve. The acute on chronic presentation is likely explained by incarceration of the inguinal component of the lipoma. Although the findings were consistent with an intermuscular lipoma extending into the inguinal canal, intraoperatively it was noted that the vascular pedicle to the lipoma arose from the deep ring. It is thus possible that the lipoma originated from preperitoneal fat (cord lipoma) and grew into the intermuscular space as suggested by the presence of the lipoma on both sides of the rectus sheath (intra-rectus space and preperitoneal space). Asymptomatic large anterior abdominal wall intermuscular lipomas may not be detected as an obvious lump, but rather as some fullness noted on straining or coughing [2]. This may be associated with a hernia, muscle aches, or shooting pains if the lipoma compresses a nerve [2].

The aetiology of lipomas is unclear, although endocrine, dysmetabolic, genetic, and traumatic theories have been suggested. A post-traumatic lipoma has been reported post-liposuction of the anterior abdominal wall and is suspected to be caused by relocation or herniation of fractured deep adipose tissue secondary to the resultant fascial injury [8]. However, these lipomas lack a capsule and are termed pseudo- lipomas, postulated to be simple adipose tissue in an abnormal location [8]. This is unlikely to be a post-traumatic lipoma because it was well encapsulated on histology findings and unrelated to her previous operation.

A good history and clinical examination are sufficient to diagnose most subcutaneous lipomas, with imaging used as an adjunct in patients with atypical symptoms and exam findings. Ultrasonography may be used to confirm the diagnosis. However, this has low accuracy and may not detect small intramuscular lipomas in larger patients [9]. A CT scan is not routinely warranted in patients with clinically incarcerated or strangulated hernias, and an urgent operative intervention should be considered with either an open or laparoscopic approach. Since our patient already had CT imaging performed prior to our review, it allowed us the benefit of planning an open approach to the inguinal hernia repair along with simultaneous excision of the intermuscular lipoma, rather than a laparoscopic approach which would have been unsuccessful in delineating and fixing the pathology.

## Conclusions

Intermuscular lipomas can be challenging to diagnose on clinical examination until they attain large sizes or present with symptoms. Uncommonly so, they may present as complications of abdominal wall hernias. A high degree of suspicion in patients reporting atypical abdominal and groin pain or abdominal wall fullness is required. As with all lipomas, the indication for excision remains the same: being for pain or patient discomfort. When an intermuscular lipoma is associated with inguinal hernias, attempting a laparoscopic total extraperitoneal approach will not likely diagnose the cause or fix the symptoms. Thus, preoperative imaging may be helpful if a patient presents with atypical symptoms and if a cough impulse is absent on clinical exam. Regardless of the operative approach, surgeons must be wary of the “tip of the iceberg” and carefully excise the lipoma in its entirety.
